# IL-4 Engagement of the Type I IL-4 Receptor Complex Enhances Mouse Eosinophil Migration to Eotaxin-1 *In* *Vitro*

**DOI:** 10.1371/journal.pone.0039673

**Published:** 2012-06-28

**Authors:** Nicola M. Heller, William M. Gwinn, Raymond P. Donnelly, Stephanie L. Constant, Achsah D. Keegan

**Affiliations:** 1 Department of Microbiology and Immunology and the Center for Vascular and Inflammatory Diseases, The University of Maryland School of Medicine, Baltimore, Maryland, United States of America; 2 Department of Microbiology, Immunology, and Tropical Medicine, The George Washington University Medical Center, Washington, District of Columbia, United States of America; 3 Division of Therapeutic Proteins, Center for Drug Evaluation and Research, U.S. Food and Drug Administration, Bethesda, Maryland, United States of America; University Hospital Freiburg, Germany

## Abstract

**Background:**

Previous work from our laboratory demonstrated that IL-4Rα expression on a myeloid cell type was responsible for enhancement of Th2-driven eosinophilic inflammation in a mouse model of allergic lung inflammation. Subsequently, we have shown that IL-4 signaling through type I IL-4 receptors on monocytes/macrophages strongly induced activation of the IRS-2 pathway and a subset of genes characteristic of alternatively activated macrophages. The direct effect(s) of IL-4 and IL-13 on mouse eosinophils are not clear. The goal of this study was determine the effect of IL-4 and IL-13 on mouse eosinophil function.

**Methods:**

Standard Transwell chemotaxis assay was used to assay migration of mouse eosinophils and signal transduction was assessed by Western blotting.

**Results:**

Here we determined that (i) mouse eosinophils express both type I and type II IL-4 receptors, (ii) in contrast to human eosinophils, mouse eosinophils do not chemotax to IL-4 or IL-13 although (iii) pre-treatment with IL-4 but not IL-13 enhanced migration to eotaxin-1. This IL-4-mediated enhancement was dependent on type I IL-4 receptor expression: γC-deficient eosinophils did not show enhancement of migratory capacity when pre-treated with IL-4. In addition, mouse eosinophils responded to IL-4 with the robust tyrosine phosphorylation of STAT6 and IRS-2, while IL-13-induced responses were considerably weaker.

**Conclusions:**

The presence of IL-4 in combination with eotaxin-1 in the allergic inflammatory *milieu* could potentiate infiltration of eosinophils into the lungs. Therapies that block IL-4 and chemokine receptors on eosinophils might be more effective clinically in reducing eosinophilic lung inflammation.

## Introduction

Eosinophils play a critical role in the pathogenesis of allergic asthma, both in human asthmatics and in mouse models of the disease [1]–[3]. They are also an important host defense mechanism against helminth parasite infection [4], [5]. Eosinophilic infiltration of the lung parenchyma and airways in response to chemotactic gradients is a characteristic feature of allergic inflammation of the lung [6]. Activated eosinophils release granules containing a wide variety of mediators that can cause tissue damage and inflammation. The T-helper (Th)2 cytokines, interleukin (IL)-4 and IL-13, are also made by these cells [7].

Interleukin-4 and IL-13 are key mediators in the development and persistence of an allergic immune response and much attention has been given to their relative roles during the process [8]. IL-4 and IL-13 exert their function by binding to IL-4 receptors on the cell surface. IL-4 binds to the interleukin-4 receptor alpha (IL-4Rα) chain, which pairs with either the common gamma chain (γC) to form type I IL-4 receptors or with the IL-13Rα1 subunit to create type II receptors. The type II receptor is also the IL-13 receptor but the ligand-binding subunit is IL-13Rα1 in this case. Human eosinophils have been shown to express IL-4Rα, both on the cell surface [9] and in pre-formed stores on the outer membrane of their granules [10]. Engagement of both types of receptor initiates activation of the signal transducer and activator of transcription (STAT)6 pathway [11] but only type I IL-4 receptors are capable of strongly activating the insulin receptor substrate (IRS)-2 pathway in myeloid cells [12]. The IRS-2 adaptor protein has the potential to trigger a variety of signaling pathways depending on the particular stimulus and cell type, including phosphoinositide 3-kinase (PI3K)/Akt, Grb/son-of-sevenless (Sos)/Rat sarcoma protein (Ras), mammalian target of rapamycin (mTOR) and protein kinase C (PKC) pathways.

The contribution of the type I and type II receptors to Th2 responses after allergen provocation and worm infection has been investigated *in vivo* using mice deficient in the IL-13Rα1 chain [13], [14]. Cellular infiltration of the lung and airspaces in response to challenge with either *S. mansoni* egg antigen [13], ovalbumin (OVA) or IL-4 [14] was essentially unaffected by the IL-13Rα1 deficiency in these mice, suggesting that signaling through type I IL-4R is essential in mediating eosinophilic inflammation. Since CC chemokine production was almost completely diminished in the IL-13Rα1-deficient mice, it was hypothesized that a type I IL-4R-induced, CC chemokine-independent pathway that can recruit eosinophils must play an important role.

IL-4 has been demonstrated to induce chemotaxis directly on eosinophils purified from human atopic donors and enhance their chemotaxis to CCL5 (RANTES, Regulated on Activation Normal T-cell Expressed and Secreted [9]). Previous work from our laboratory demonstrated that IL-4Rα expression on a myeloid cell type enhanced Th2-driven eosinophilic inflammation in an OVA sensitization and challenge mouse model of allergic lung inflammation [15]. Given the importance of the eosinophil in allergic inflammation, we investigated whether mouse eosinophils responded directly to IL-4 and IL-13 *in vitro* and how these cytokines affected the chemotactic function of the cells. We found that mouse eosinophils express both type I and type II receptors on their surface and mRNA for both type I and II receptor subunits. IL-4, but not IL-13, promoted strong tyrosine phosphorylation of STAT6 and both cytokines induced tyrosine phosphorylation of IRS-2. Phosphorylation of other signaling intermediates [AKT, p38, extracellular-regulated kinase (ERK)1/2] after IL-4/IL-13 stimulation was not detected in these cells. IL-4 was able to enhance eosinophil migration to CC chemokines better than IL-13. Pre-treatment of mouse eosinophils with IL-4 but not IL-13 enhanced migration to eotaxin-1. This enhancement of migration was dependent on expression of the type I IL-4 receptor, as eosinophils deficient in the γC subunit did not show this IL-4-enhanced migratory capacity. These data suggest that mouse eosinophils are more responsive to IL-4 than IL-13 and the presence of this cytokine in combination with CC chemokines, as is found in an allergic setting *in vivo*, would potentiate the infiltration of eosinophils into lung tissue.

## Materials and Methods

### Mice and Reagents

All experiments were conducted under the approval of the University of Maryland Baltimore Institutional Animal Care and Use Committee. IL-5 transgenic (Tg) mice on a C57BL/6 background [16] were provided by Dr James Lee at the Mayo Clinic, Scottsdale, AZ and were maintained as described [17]. IL-5 transgene-positive mice were identified by PCR of ear punch samples as described [16]. Mice deficient for γC (B6.129S4-Il2rg^tm1Wjl^/J) and wildtype (WT) C57BL/6 were purchased from the Jackson Laboratories, Bar Harbor, ME. The mouse macrophage cell line, RAW 264.7, was purchased from the ATCC (Manassas, VA). IL-4 and IL-13 were purchased from R & D Systems (Minneapolis, MN) and all chemokines, IL-5, stem cell factor (SCF) and Flt3-ligand (Flt3-L) were purchased from Peprotech (Rocky Hill, NJ).

### Isolation of Mouse Eosinophils

**Figure 1:**
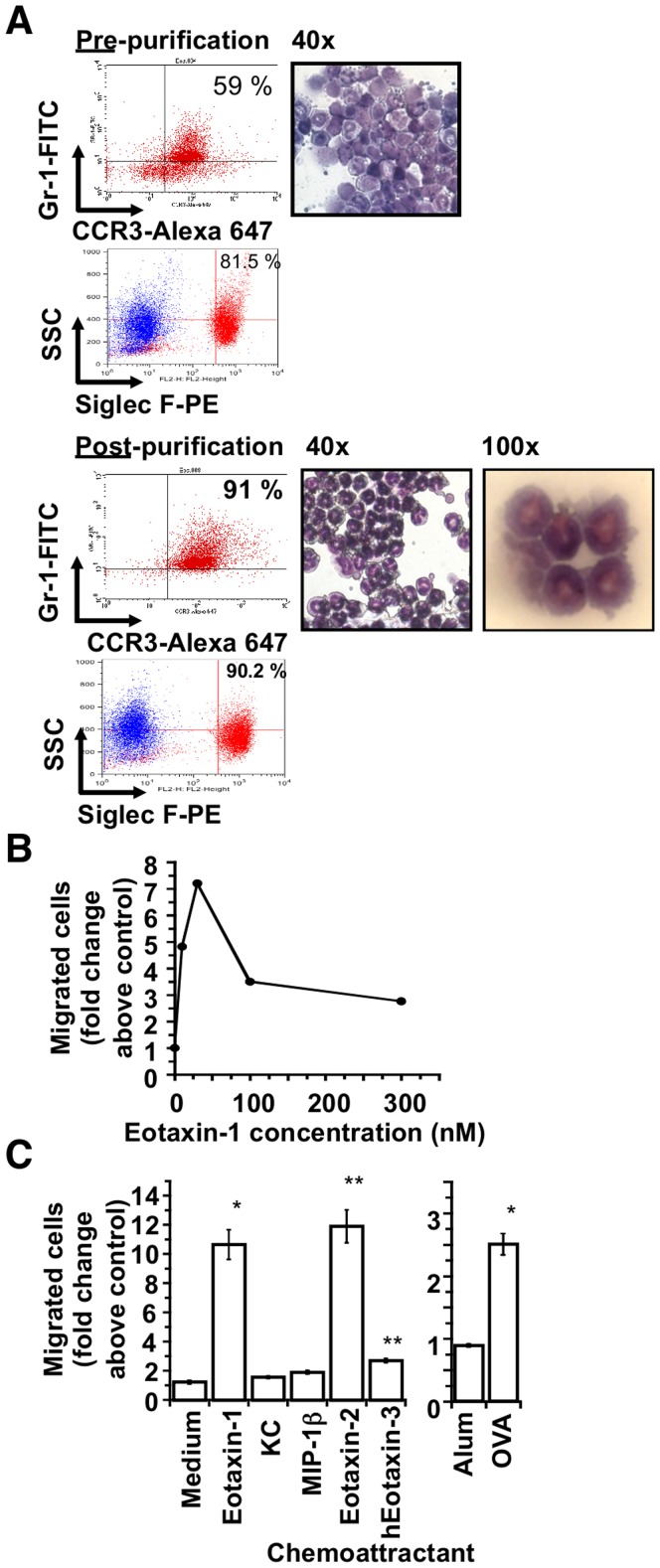
Purification and characterization of mouse eosinophils from IL-5 Tg mice. (A). FACS and cytospin analysis of peripheral blood “buffy coat” cells prior to and after MACS column purification. Peripheral blood from IL-5 Tg mice was spun over Percoll, the red cells were lysed and the eosinophils were purified using a negative separation MACS column-based strategy as described [17] with modifications [18]. The purity of the eosinophil preparation was assessed by FACS double-staining for the surface markers, Gr-1 and CCR3, as well as Siglec F and by examination of cytospins following DiffQuik staining (40×). Red-stained eosinophilic granules are clearly visible in the cells at 100× magnification. (B). Eosinophils were purified as described in the Methods. Eosinophils (5 × 10^5^ per Transwell insert) were placed into the upper chamber of a 24-well Transwell chemotaxis assay plate in duplicate with increasing concentrations of eotaxin-1 in the lower chamber and incubated at 37°C for 2 hours [18]. The total number of live cells that had migrated into the bottom chamber at each concentration was counted and divided by the number that migrated to medium alone to give the fold change above control, which was plotted against eotaxin-1 concentration. (C). Migration of mouse eosinophils to CCR1, CCR3, CCR4 agonists and BALF from Alum- and OVA/Alum-sensitized and challenged mice. Thirty nanomolar solutions of each chemokine or BALF from mice subjected to ovalbumin sensitization and challenge or alum control diluted 1∶4 [15] were used in the chemotaxis assay as described in (B) in triplicate. Migrated cells were collected after 2 hours and counted by FACS with CountBright™ beads as described in the Methods. (*P<0.05, compared to medium control or Alum-treated control).

Mouse eosinophils constitute about 60% of the leukocytes in the peripheral blood of IL-5 Tg mice [16] and they were purified from peripheral blood of IL-5 Tg mice following cardiac puncture as described [17], [18]. Briefly, the buffy coat layer from Percoll E-separated blood [18] was subjected to red cell lysis with 0.2% NaCl to minimize granule alterations [19] and negative selection with magnetic anti-CD90 (Thy 1.2) and anti-CD45R (B220) microbeads (Miltenyi Biotec, Auburn, CA) as per the manufacturer’s instruction. The purity of the eosinophils was determined by double-staining with anti-CC chemokine receptor (CCR)3, either in combination with anti-Gr-1 (both from BD Biosciences) or anti-Siglec F antibodies (BD Biosciences) by fluorescence-activated cell sorting (FACS) and also by DiffQuik staining (Dade Boehringer AG) following cytospin (Figure 1A). Preparations were routinely >90% CCR3/Gr-1 double-positive, and >90% Siglec F-positive and the mouse eosinophils had a typical ring-shaped nuclear morphology and red granules by microscopic evaluation (Figure 1A). The viability of the cells after purification was >97% by propidium iodide exclusion.

Eosinophils were also generated by differentiation of bone marrow harvested from γC^+/+^ or γC^−/−^ mice using stem cell factor (SCF), Flt3-ligand (Flt3-L) and IL-5 as described [20]. Briefly, bone marrow was harvested, red cells were lysed and the cells were cultured in SCF and Flt-3L for four days, followed by culture in IL-5 for an additional ten days. Similar numbers of eosinophils were generated from both strains after the fourteenth day of culture. Cell viability was >78% by Trypan blue exclusion and the eosinophils from both strains were >76% Siglec F/Gr-1 double-positive by FACS analysis. After day 14, the cells were harvested and washed in RPMI containing 5% bovine serum albumin (Sigma). Chemotactic responses were evaluated as described below. Eosinophils derived from γC^+/+^ or γC^−/−^ mice migrated similarly in response to eotaxin-1, indicating no defect in chemokine-elicited migration.

#### Allergic lung sensitization

Mice were sensitized and challenged with chicken egg ovalbumin using a modified protocol described by Wang *et al* [21]. Each mouse was immunized with either 100 µg of OVA/alum or alum alone on day 1 and again on day 6. After the last sensitization step, mice were challenged with aerosolized 1% OVA in phosphate-buffered saline (PBS) for 40 minutes each day on days 12 and 14 and BALF was collected 48 hours after the last OVA challenge. The BALF was collected by lavaging the lungs with 1 ml PBS and cell-free supernatant was isolated and stored at –70°C until use. BALF from three mice in each treatment group was pooled and diluted 1∶4 in RPMI/5% BSA before being added to the bottom chamber of the Transwell plate.

### Transmigration Assay

Chemotaxis of purified eosinophils was performed as described [17] with modifications [18]. A typical Transwell assay (Costar, 6.5 mm diameter, 5 µm pore size) was used. The eosinophils prepared as described above were washed and resuspended in RPMI containing 5% bovine serum albumin (Sigma). Five×10^5^ cells in 200 µL were added to the top chamber and 500 µL of recombinant mouse chemokine, bronchoalveolar lavage fluid (BALF) harvested from OVA-primed mice (described above), IL-4, or IL-13 were added to the bottom chamber. The eosinophils were allowed to migrate for two hours at 37°C in the tissue culture incubator. For experiments involving pre-treatment of eosinophils with cytokines, purified cells were incubated for 30 minutes at 37°C with increasing concentrations of IL-4 or IL-13. Pre-treated cells were then either added directly to the Transwell chamber or washed to remove cytokine. The number of migrated eosinophils was evaluated by FACS using CountBright™ Absolute counting beads (Invitrogen/Molecular Probes) as per the manufacturer’s instructions. Migration is expressed as the fold change in migration. Fold change was calculated as the number of cells that migrated in response to a stimulating agent divided by the number that migrated in response to medium alone. The viability of migrated cells after the chemotaxis assay by propidium iodide exclusion was ≥91%.

### Signaling Analysis

Purified eosinophils were serum-starved in RPMI 1640 at a density of 10×10^6^ cells/mL for two hours prior to signaling analysis. Cells were then stimulated with eotaxin 1, IL-4, or IL-13 for 15 min at 37°C. Cells were then washed in ice-cold PBS containing 1 mM sodium orthovanadate, pelleted and lysed in lysis buffer [12] with additional 100 nM calyculin A and 5 mM caproic acid. Lysates were analyzed by Western blot for phosphorylated STAT6^Tyr641^, AKT^Ser473^, p38^Thr180/Tyr182^ and ERK1/2^Thr202/Tyr204^ and α-tubulin as described [12]. Tyrosine phosphorylation of IRS-2 was analyzed by immunoprecipitation with antibodies to IRS-2 followed by Western blot with anti-phosphotyrosine antibodies.

### Receptor Analysis

Purified eosinophils were stained with specific antibodies to mouse IL-4Rα (BD Biosciences), γC (BD Biosciences) or IL-13Rα1 and were analyzed by FACS as described previously [12]. The anti-mouse IL-13Rα1 monoclonal antibody was developed in the laboratory of Ray Donnelly against a peptide in the D1 domain of the mouse IL-13Rα1 protein [22]. This antibody binds specifically to the IL-13Rα1 chain and does not cross-react with IL-4Rα, γC or IL-13Rα2. After washing away unbound anti-mouse IL-13Rα1 antibody, cells were incubated with an anti-mouse-phycoerythrin antibody and fluorescence was analyzed by FACS. Expression of the mRNA for each of the type I and type II receptor components was determined by quantitative RealTime PCR as follows. Total RNA was isolated from purified eosinophils or from the macrophage cell line RAW264.7 as a positive control using the RNeasy kit (Qiagen) and converted to cDNA with the SuperScript III RTase kit (Invitrogen), both according to the manufacturer’s instructions. RealTime PCR was performed with the following primer sets: hypoxanthine phosphoribosyltransferase *(Hprt)* forward, 5′-GCTGACCTGCTGGATTACATTAA-3′ and *Hprt* reverse5′-TGATCATTACAGTAGCTCTTCAGTCTGA-3′, *Il-4rα* forward, 5′-ATCTGCGTGCTTGCTGGTTCT-3′ and *Il-4rα* reverse, 5′-CTGGTATCTGTCTGATTGGACCG-3′, *γC* forward, 5′-GTGCAGTACCGGAGCAAC AGA-3′ AND *γC* reverse, 5′-AAATGTGTACCGTTTCAGCTCATC-3′, *Il-13rα1* forward, 5′-CATCTTCTCCTCAAAAATGGTGCC-3′, *Il-13rα1* reverse, 5′-GGATTATG ACTGCCACTGCGAC-3′, *IL-13rα2* forward, 5′-CACACCTGGAGGACCCATTC-3′ and *Il-13rα2* reverse, 5′-GTGGCAGACTCCCAGGAAATAT-3′. The quantitation of the mRNA by RealTime PCR from duplicate wells is expressed using relative quantitation, expressed in the standard format of 2^–ΔΔCt^. The relative expression of receptor subunit mRNA from mouse eosinophils was compared to the amount of expression in the RAW cell line, the positive control (2^–ΔΔCt^  = 1). The PCR products were also resolved on a 3% agarose gel and visualized by ethidium bromide staining.

### Statistical Analyses

Data were analyzed by Student t-test or one-way ANOVA with Fisher’s least significant difference *post-hoc* test as appropriate. Differences between groups were considered significant when *P*<0.05.

## Results

### IL-4 Receptor Expression on Mouse Eosinophils

Eosinophils isolated from IL-5 transgenic mice were greater than 91% pure based on FACS analysis with CCR3/Gr-1/Siglec F and appeared morphologically normal by microscopic examination following cytospin and DiffQuik staining (Figure 1A). The mouse eosinophils were TER119, CD16 and CCR5-negative and had high side scatter by FACS. To characterize the responsiveness of these purified eosinophils, a concentration-response analysis of eosinophil migration to eotaxin-1 was performed (Figure 1B). The maximum migration was observed at 30 nM eotaxin-1 (252 ng/mL), with less migration at higher eotaxin-1 concentrations due to desensitization typical of G-protein-coupled chemokine receptors at high ligand concentrations. Therefore, 30 nM eotaxin-1 was used in subsequent chemotaxis experiments. We also verified the ability of our purified cells to migrate to a variety of different recombinant mouse chemokines. To compare the migration of our purified eosinophils to 30 nM eotaxin-1 to other mouse CC and CXC chemokines, equimolar concentrations (30 nM) of various mouse CC and CXC chemokines were placed in the bottom chamber and migration of eosinophils was measured after two hours (Figure 1C). Mouse eosinophils migrated best to the CCR3 ligands, eotaxin-2 and -1 as has been previously described [17]. Migration of the cells to BALF from OVA-challenged mice was also significantly elevated above the migration to BALF from control mice treated with alum. Migration to the other ligands (KC, macrophage inflammatory protein (MIP)1β and human eotaxin-3) was not significantly different than migration to medium alone, as was observed previously [17].

**Figure 2:**
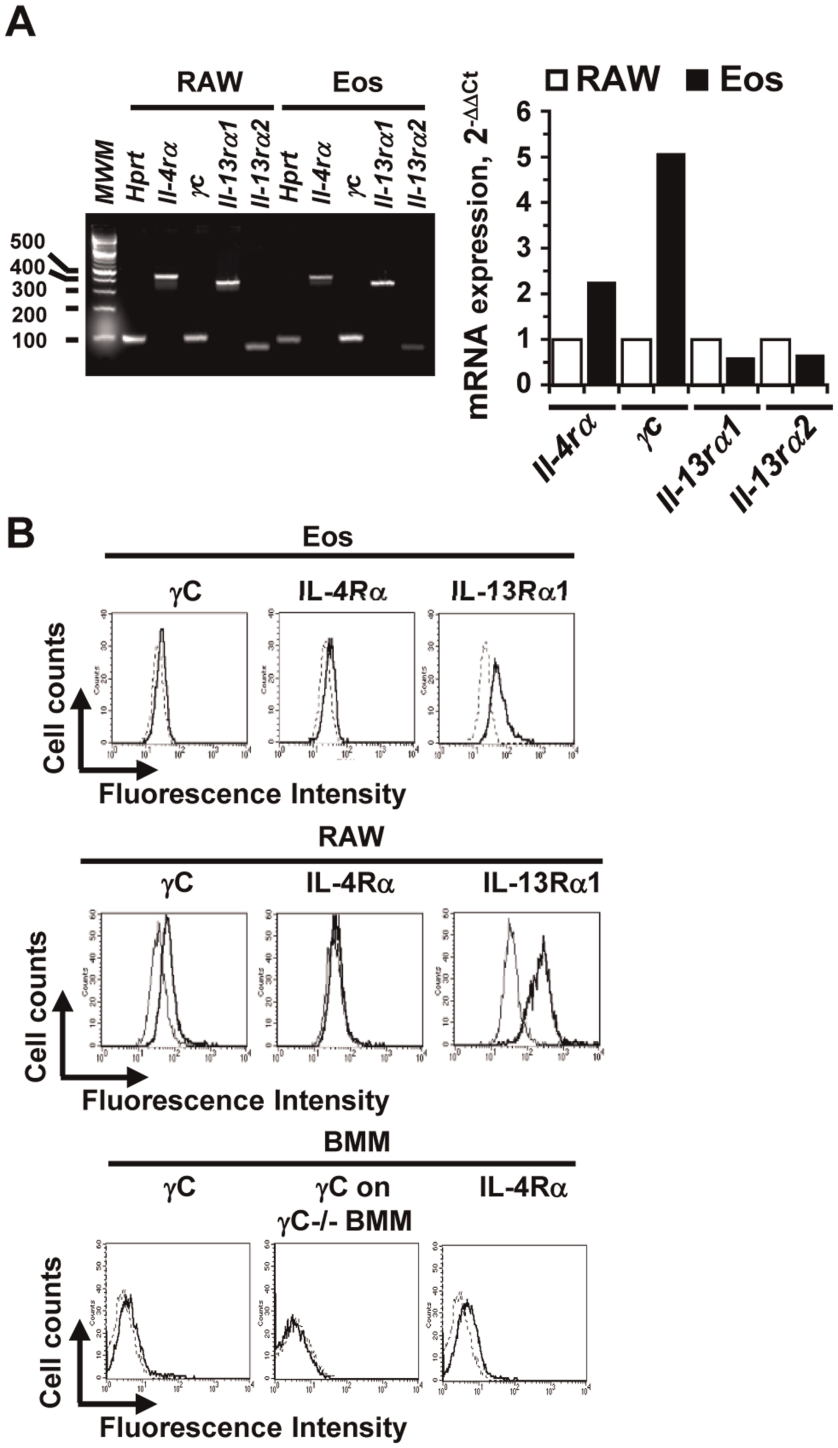
Expression of mRNA and surface protein for components of the IL-4/IL-13 receptor complex on mouse eosinophils. (A). Expression of mRNA for the different subunits of the IL-4/IL-13 receptor complex. RNA was extracted from purified eosinophils and the mouse macrophage cell line, RAW 264.7, and cDNA was synthesized. Relative quantitation by RealTime PCR with specific primer pairs for each receptor subunit was performed, using the standard 2^−ΔΔCt^ method, and the products were resolved on a 3% agarose gel. The amount of expressed mRNA for each subunit from mouse eosinophils (filled bars) relative to the amount in the control RAW cell line ( = 1, open bars) was graphed. (B). Cell surface expression of the IL-4/IL-13 receptor subunits on mouse eosinophils. FACS analysis using specific antibodies to each receptor subunit was performed. Representative FACS histograms from one experiment (of four to five independent experiments) are shown with staining with specific antibody (solid black line) and isotype-matched control antibody (dotted line). Receptor staining on mouse bone marrow-derived macrophages from wildtype and γC-deficient mice, generated as described [12], and to RAW 264.7 cells is shown for comparison.

RealTime PCR and FACS analysis was used to determine the expression of mRNA and surface protein for the different components of the IL-4 and IL-13 receptor complexes. Mouse eosinophils express mRNA for the common γ chain (γC), IL-4Rα, IL-13Rα1 and IL-13Rα2 as determined by RealTime PCR (Figure 2A). Mouse eosinophils also express, γC, IL-4Rα and IL-13Rα1 protein on the cell surface as detected by FACS (Figure 2B), similar to the surface expression pattern seen on human eosinophils by Myrtek *et al* [23]. The expression of γC and IL-4Rα chain on primary mouse bone marrow-derived macrophages (BMM) isolated from γC^+/+^ or γC^−/−^ and on the mouse macrophage cell line, RAW 264.7, is shown for comparison to the purified mouse eosinophils. Expression of these receptor components on primary eosinophils was similar to expression on primary BMM. These results are consistent with equilibrium binding studies on primary cells showing that the numbers of IL-4 and IL-13 binding sites range from 50–5000 per cell [24]. These results indicate that eosinophils express all the receptor subunits to form both type I and type II receptors.

**Figure 3:**
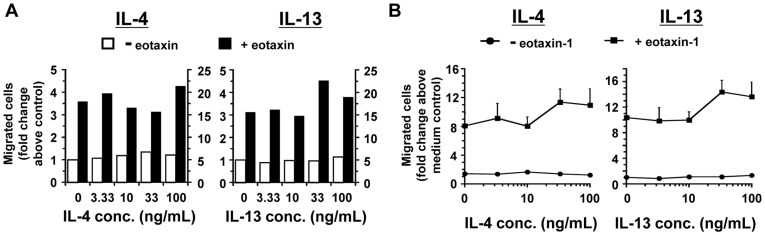
Migration of purified mouse eosinophils to IL-4 and IL-13 in the presence or absence of eotaxin-1. (A). Eosinophil chemotaxis to IL-4 (left panel) and IL-13 (right panel) in the presence (filled bars) or absence (open bars) of 30 nM eotaxin-1 was measured. The indicated concentrations of IL-4 were placed in the lower chamber and 5 x 10^5^ eosinophils were placed in the upper chamber. A representative experiment is shown. (B). Average data from multiple independent chemotaxis experiments performed as in (A) using IL-4 or IL-13 with (squares) or without (circles) eotaxin in the lower chamber (*n* = 2–4). For the IL-4 graph, number of migrated cells to medium = 9,733± SEM 2,468 and to eotaxin-1 = 54,444± SEM 10,926 and for the IL-13 graph, number of migrated cells to medium = 4,478± SEM 689 and to eotaxin-1 = 56,654± SEM 4,949.

**Figure 4:**
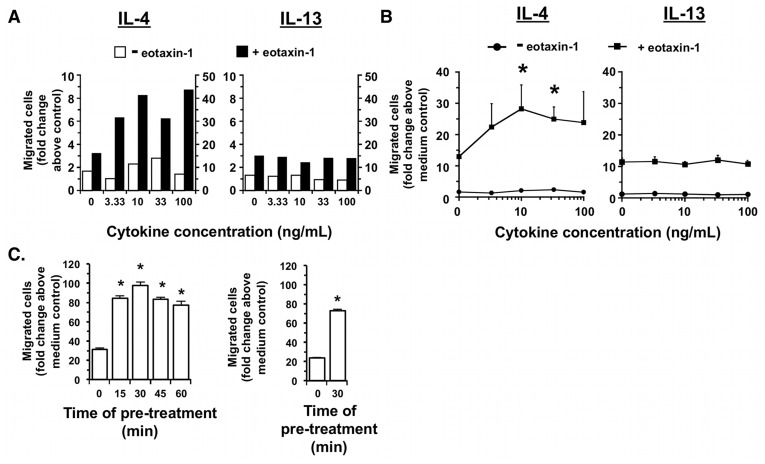
Pre-treatment with IL-4 but not IL-13 enhanced migration of purified mouse eosinophils to eotaxin-1 and eotaxin-2. Eosinophils were purified as described in the Methods. (A). Eosinophils were then incubated with various concentrations of IL-4 or IL-13 at 37°C for 30 minutes. The treated cells were washed and then placed (5×10^5^) into the upper chamber insert of the Transwell plate; the lower chamber contained either 0 or 30 nM eotaxin-1. The chemotaxis assay was performed and the number of migrated cells was counted as described in Figure 1. A representative experiment is shown. (B) Average data from three independent chemotaxis experiments (*n* = 3; *P<0.05, compared to migration to eotaxin-1 alone). For the IL-4 graph, number of migrated cells to medium = 11,614± SEM 3,257 and to eotaxin-1 = 102,435± SEM 27,964 and for the IL-13 graph, number of migrated cells to medium = 8,581± SEM 2,163 and to eotaxin-1 = 75,651± SEM 20,436. (C, left panel) Time of IL-4 pre-treatment of eosinophils prior to chemotaxis. Eosinophils were pre-treated with 10 ng/mL IL-4 for the indicated times, subjected to chemotaxis to 30 nM eotaxin-1 and counting as in (A). *P*<0.05. (C, right panel) Eosinophils were pre-treated with 10 ng/mL IL-4 for the indicated time, subjected to chemotaxis to 30 nM eotaxin-2 and counting as in (A). *P*<0.05.

### IL-4 Pre-treatment Enhanced Chemotaxis of Mouse Eosinophils to Eotaxin-1

Two earlier studies noted that the cytokines IL-4 and IL-13 could induce chemotaxis in human eosinophils but with conflicting results. Dubois *et al* described migration to IL-4 (10^−8^ to 10^−10^ M) of human eosinophils derived from individuals with atopic dermatitis and allergic asthma but not normal controls [25]. In contrast, concentrations of ≥60 ng/mL IL-13 but not IL-4 were found to be chemotactic for human eosinophils purified from normal volunteers [26]. Intraperitoneal or intradermal injection of mice with IL-4 induced eosinophilic infiltration [27]. Whether mouse eosinophils respond directly to IL-4 or IL-13 *in vitro* was not known. Therefore, we used primary mouse eosinophils in an *in* *vitro* Transwell chemotaxis assay to test migration to ng/mL concentrations of recombinant mouse IL-4, IL-13 or 30 nM eotaxin-1 alone or in the presence or absence of IL-4 or IL-13.

We found that neither IL-4 nor IL-13 induced migration of eosinophils or enhanced migration of mouse eosinophils to 30 nM eotaxin-1 when they were placed in the bottom chamber in increasing concentration (Figure 3A, representative data from one experiment and 3B, mean data from multiple experiments). There was a trend to enhancement of migration compared to eotaxin-1 alone at the higher concentrations of cytokine although statistical significance was not reached. Because we were concerned that the eosinophils were at maximum migratory capacity at 30 nM eotaxin-1 and we may be missing any enhancing effects of the addition of the cytokines, we repeated the chemotaxis experiments using a lower dose of eotaxin-1. When a suboptimal concentration (10 nM) eotaxin-1 was used in the bottom chamber with either IL-4 or IL-13, the modest enhancement of eosinophil migration compared to 10 nM eotaxin-1 alone was not greater than that observed at 30 nM eotaxin-1 in the presence or absence of cytokine (data not shown). However, we found that pre-treatment of the mouse eosinophils for 30 minutes with IL-4, but not with IL-13, augmented the chemotactic response of the mouse eosinophils to eotaxin-1 (Figure 4). A representative experiment directly comparing the effect of increasing concentrations of IL-4 and IL-13 pre-treatment on migration to eotaxin-1 is shown (Figure 4A). In multiple independent experiments, pre-treatment with IL-4 significantly enhanced migration to eotaxin-1 (left panel, squares) while pre-treatment with IL-13 (right panel, squares) did not (Figure 4B). Migration was not enhanced at a suboptimal concentration of eotaxin-1 (10 nM) in the bottom chamber following cytokine pre-treatment compared to 10 nM eotaxin-1 alone (data not shown). The effect of the time of IL-4 pre-treatment (10 ng/mL) on migration of eosinophils to 30 nM eotaxin-1 was also tested (Figure 4C, left panel). A 30 minute pre-treatment of the cells with IL-4 enhanced migration to eotaxin-1 optimally. Migration of eosinophils to 30 nM eotaxin-2 was also enhanced to a similar degree (approximately three-fold) by pre-treatment with IL-4 (Figure 4C, right panel).

**Figure 5:**
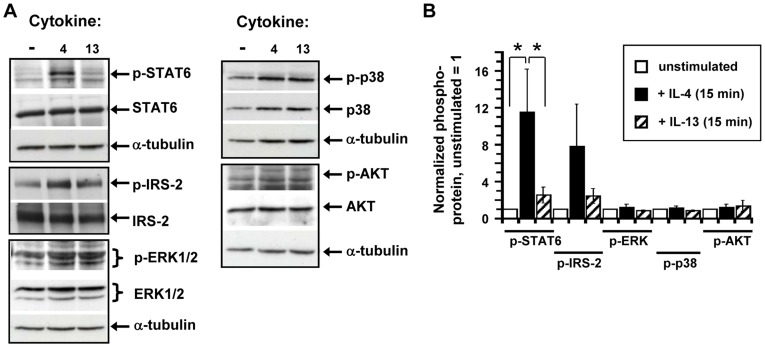
Signaling responses to IL-4 and IL-13 in mouse eosinophils. (A) Eosinophils were purified as described in the Methods. The cells were serum-starved for 2 hours prior to stimulation with 20 ng/mL IL-4 (“4”) or IL-13 (“13”) for 15 mins or no stimulation (“–”). Eosinophil cell lysates were prepared as described in the Methods and subjected to SDS-PAGE and Western blotting with antibodies specific for the phosphorylated form of the particular signaling protein. Blots were stripped and re-probed with antibodies specific for the unphosphorylated form of the protein. Films from one representative experiment are shown. (B). Densitometric analysis of Western blot films. Films from independent experiments were scanned and the amount of phosphoprotein/total target protein was quantitated by densitometry. This ratio was normalized for loading (to α-tubulin) and graphed with the amount of phosphoprotein in unstimulated cells equal to 1). Average data from three to four independent experiments are shown (*P<0.05).

**Figure 6:**
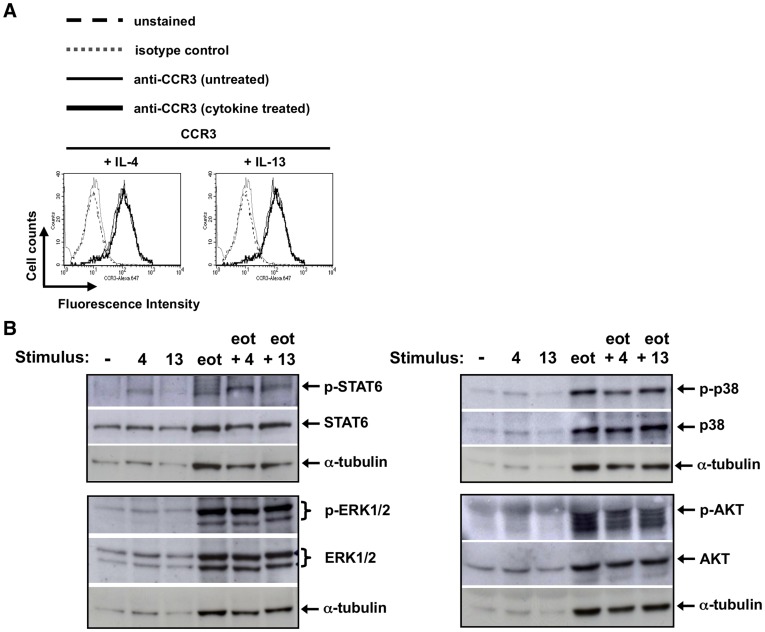
Effect of pre-treatment with IL-4 and IL-13 on CCR3 expression and eotaxin-1 signaling in mouse eosinophils. (A). Eosinophils were treated with (heavy solid line) or without (light solid line) 100 ng/mL IL-4 (left panel) or IL-13 (right panel) for 30 min and FACS analysis with specific antibodies to CCR3 (solid lines), an isotype control (dotted) or unstained (dashed line) was performed as described (Figure 1). (B) The cells were serum-starved for 2 hours prior to stimulation with 20 ng/mL IL-4 (“4”) or IL-13 (“13”) for 15 mins or 30 nM eotaxin-1 (“eot”) for 1 minute or no stimulation (“–”) as indicated. Cell lysates were prepared and analyzed by Western blotting as Figure 5.

### IL-4 and IL-13-induced Signaling in Mouse Eosinophils

Previous data from our lab demonstrated that IL-4 and IL-13 can exert differential activation of signaling molecules downstream of the IL-4 receptor complex, in particular on the IRS-2 pathway, despite the fact that type I and type II receptors share a common subunit, the IL-4Rα chain. Because pre-treatment with IL-4 enhanced chemotaxis while IL-13 did not, we reasoned that the two cytokines may have differential effects on signaling pathways. To this end, a variety of signaling pathways were analyzed by Western blotting of eosinophil lysates after stimulation with IL-4 and IL-13 (Figure 5). The IL-4-induced tyrosine phosphorylation of STAT6 in the mouse eosinophils was approximately ten-fold above unstimulated (*P*<0.05) but IL-13 induced phospho-STAT6 only two-fold above the unstimulated signal at the equivalent cytokine concentration (Figure 5B). This contrasts with our observations in mouse BMM where stimulation with high concentrations of IL-4 and IL-13 induced equivalent amounts of phospho-STAT6 [12]. Both cytokines stimulated tyrosine phosphorylation of IRS-2, with IL-4 again inducing a more robust response than IL-13. The IL-4-induced phosphorylation of IRS-2 was six-fold of the unstimulated amount while the IL-13-induced signal was two-fold over background, consistent with our previous observations in mouse BMM [12]. Neither cytokine elicited significant phosphorylation of ERK1/2, p38 or AKT relative to unstimulated eosinophils. This finding contrasts with observations that IL-4 elicited p38 phosphorylation in human eosinophils [28].

To determine whether the increased migration to eotaxin-1 following IL-4 stimulation might be due to increased surface expression of the eotaxin-1 receptor, CCR3, on the eosinophils, we analyzed the CCR3 expression on the cell surface by FACS in IL-4- and IL-13-pre-treated cells compared to unstimulated cells (Figure 6A). There was little change in the cell surface expression of CCR3 following stimulation with IL-4 or IL-13 (at 1, 10 or 100 ng/mL IL-4 or IL-13), possibly because CCR3 expression on eosinophils derived from IL-5 Tg mice is already elevated approximately three-fold above that measured on wildtype eosinophils [29].

**Figure 7:**
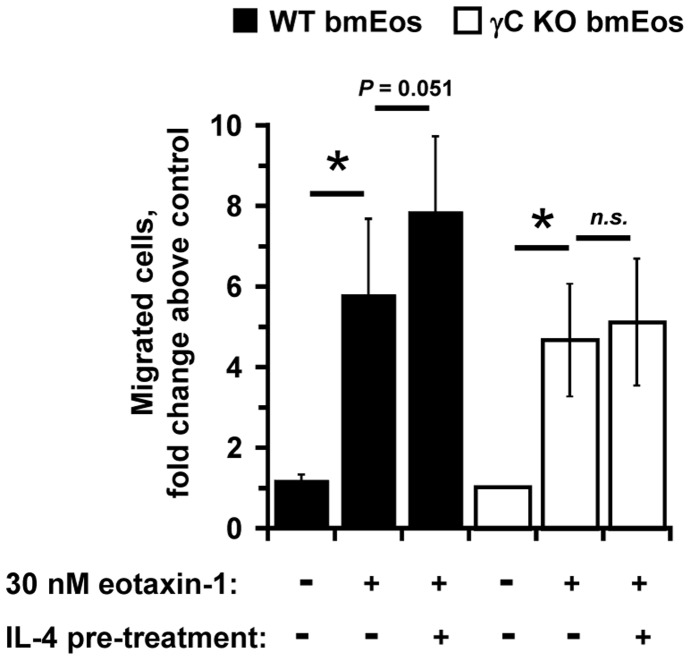
Migration of bone marrow-derived eosinophils (bmEos) from γC^+/+^ and γC^−/−^ mice to eotaxin-1. Eosinophils were differentiated from the bone marrow of γC^+/+^ (black bars) or γC^−/−^ (open bars) mice *in vitro* as described in the Methods. The cells were either untreated or primed with 10 ng/mL IL-4 for 30 min prior to placement into the upper chamber of the Transwell chemotaxis assay. Eotaxin-1 (30 nM) was placed in the lower chamber. Migrated eosinophils from triplicate wells were counted as described in Figure 1. The average data from two experiments performed in triplicate are shown (* *P*<0.05, compared to medium control). For γC^+/+^, mean number of cells migrated to medium = 44,567± SEM 10,947; to eotaxin-1 = 155,817± SEM 34,864 and IL-4 pre-treated cells to eotaxin-1 = 217,043± SEM 24,531. For γC^−/−^, mean number of cells migrated to medium  = 51,640± SEM 12,840; to eotaxin-1 = 149,862± SEM 13,133 and IL-4 pre-treated cells to eotaxin-1 = 145,768± SEM 19,289.

**Figure 8:**
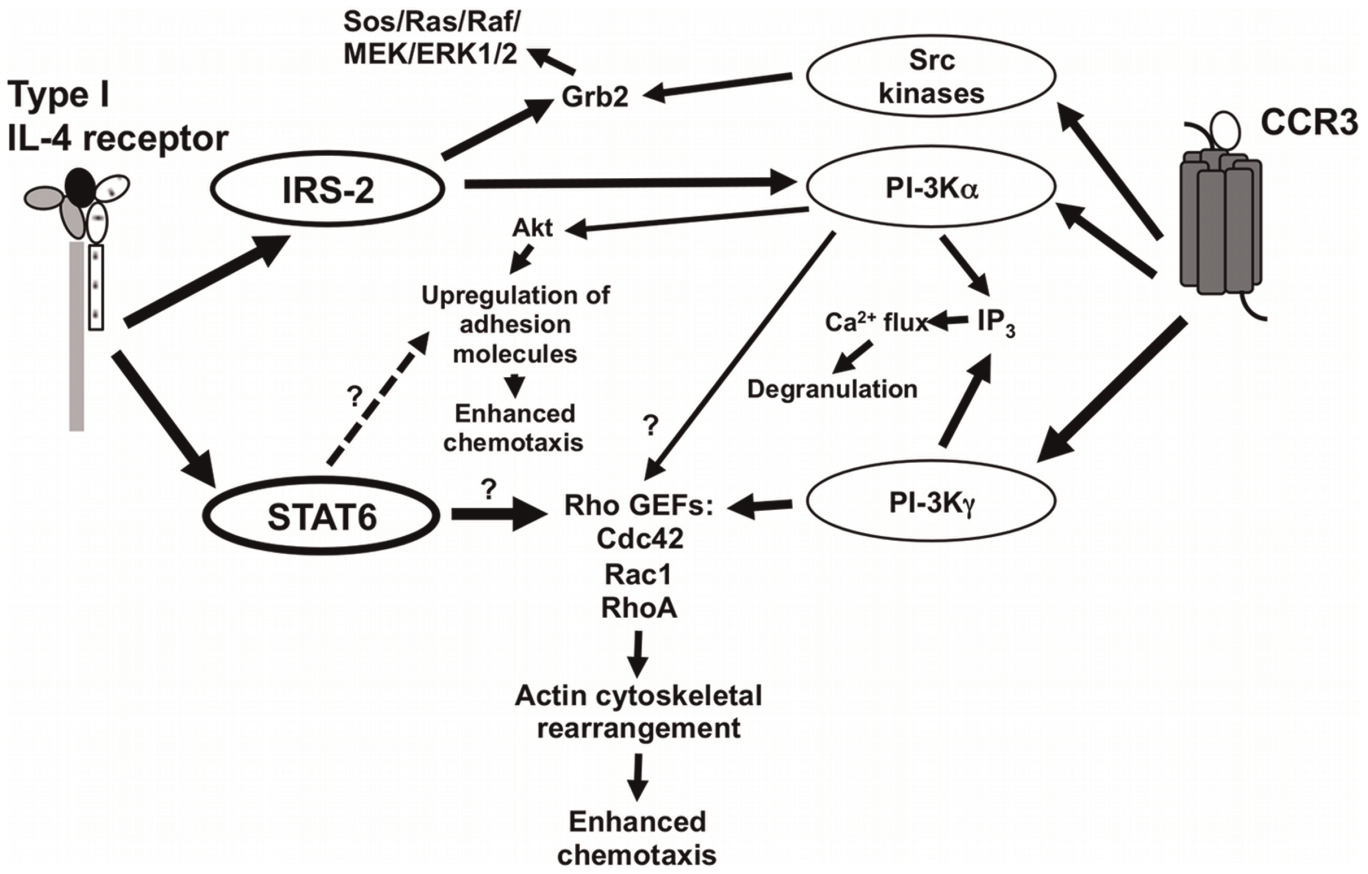
Proposed mechanism of IL-4 enhancement of mouse eosinophil chemotaxis to eotaxin. Our data showed that the STAT6 and IRS-2 pathways are activated in mouse eosinophils in response to IL-4 (black oval) but to a lesser degree by IL-13. Engagement of CCR3 by eotaxin-1 (white oval) activates G-proteins leading to activation of PI3K isoforms and other signaling pathways. The two pathways may intersect to enhance migration in the activation of Rho guanine nucleotide exchange factors (GEFs), which leads to cytoskeletal rearrangement and enhanced chemotaxis. IL-4/STAT6 has been implicated in spreading/movement of B-cells via Rho GEFs [63]. IL-4 is also known to upregulate adhesion molecules involved in extravasation/chemotaxis – this may be due to activation of STAT6-mediated transcription or activation of the AKT pathway downstream of IRS-2.

Although there was no change in the amount of CCR3 expressed on the cell surface following incubation with IL-4, we tested whether IL-4 augmented eosinophil signaling responses to eotaxin-1 downstream of the chemokine receptor (Figure 6B). Eotaxin-1 induced the phosphorylation of ERK1/2, as anticipated from studies of human eosinophils [30]–[32]. However, IL-4 did not augment eotaxin-1-induced ERK phosphorylation in the mouse cells and neither did IL-13. Eotaxin-1 stimulation of mouse eosinophils induced a small amount of phosphorylation of p38 and AKT on Ser473, as has been described for stimulation of human eosinophils with eotaxin [32] and other chemoattractants [30]. Neither treatment with IL-4 nor IL-13 in combination with eotaxin-1 resulted in a greater amount of the phosphorylated forms of STAT6, p38 or AKT compared to stimulation with eotaxin-1 alone.

### The Role of type I IL-4 Receptor in Cytokine-induced Enhancement of Chemotaxis

Since IL-4 enhanced chemotaxis to eotaxin-1 while IL-13 did not, we hypothesized that the enhancement was activated specifically by the type I receptor, the receptor complex unique for IL-4. To test this hypothesis, chemotaxis studies were performed using eosinophils lacking expression of γC, which is required to form functional type I IL-4 receptor complexes. Eosinophils were prepared *in vitro* by culturing bone marrow isolated from γC^+/+^ or γC^−/−^ mice with stem cell factor, Flt3-L and IL-5 (bmEos, [20]. Equal numbers of viable eosinophils that were 74% CCR3/Gr-1-double positive and 78% and 76% positive for Siglec-F were obtained from γC^+/+^ and γC^−/−^ bone marrow respectively. Expression of γC, IL-4Rα and IL-13Rα1 on both γC^+/+^ or γC^−/−^ bone marrow-derived eosinophils showed a similar pattern to that seen on eosinophils purified from the IL-5 Tg mice (data not shown), with the exception of the γC subunit that was absent on the bmEos derived from the γC-deficient animals, as expected. Surface expression of IL-13Rα2 could not be evaluated due to lack of available reagents for mouse cells and quantitative PCR is not a reliable measure of protein expression at the cell surface.

We found that eotaxin-1 induced migration of γC^+/+^ and γC^−/−^ bmEos by four to six-fold (Figure 7). This level of chemotaxis was lower than that observed using eosinophils prepared from the peripheral blood of IL-5 Tg mice (which was typically eight to ten-fold above medium control, Figure 1). This observation is consistent with previous studies of bmEo migration to eotaxin-1, (2.25-fold above medium control in response to 100 ng/mL eotaxin-1, [20]). We chose to pre-treat the bmEos with 10 ng/mL IL-4 as this pre-treatment concentration elicited maximum migration of the purified peripheral blood eosinophils. Pre-treatment of the γC^+/+^ bmEos with IL-4 enhanced migration to eotaxin-1, compared to migration of the untreated cells to eotaxin-1 alone (*P*  = 0.051). In contrast, IL-4 pre-treatment clearly did not enhance chemotaxis of γC^−/−^ bmEos in response to eotaxin-1. These data indicate that the type I IL-4 receptor is required for the IL-4-induced enhancement of eosinophil migration induced by eoxtaxin-1.

## Discussion

Eosinophilic infiltration of the airways is characteristic of an allergic inflammatory response in the lung and migration of these cells from the blood to the airways occurs as a result of chemokine gradients secreted by lung resident cells, such as the airway epithelium. *In vitro* experiments using purified human eosinophils have demonstrated chemotactic movement to both Th2-type cytokines, IL-4 [25], [33] and IL-13 [26]. Overnight incubation with IL-4 enhanced the migration of human eosinophils to RANTES [9]. Human eosinophils express the γC chain of the type I IL-4 receptor on their surface and expression of IL-4Rα is constitutive [9]. A previous report from our laboratory demonstrated that non-lymphoid, bone marrow-derived cell(s) that expresses the IL-4Rα chain contributed to the severity of lung inflammation and mucus production in a mouse model of an ovalbumin-induced allergic lung inflammation [15]. Careful characterization of the expression pattern of IL-4/IL-13 receptors on mouse eosinophils and how the signaling and chemotactic responses of mouse eosinophils are affected by these Th2 cytokines is unknown. In our study, we found that mouse eosinophils express the γC, IL-4Rα, and IL-13Rα1 receptor chains on their surface, and that pre-treatment with IL-4 but not IL-13 enhanced cell migration to eotaxin-1. The enhancement of chemotaxis following pre-treatment with IL-4 was not due to induction of CCR3 on the surface of the eosinophils, confirming earlier studies of modulation of CCR3 expression on human eosinophils by various cytokines [34]. We found that the enhancement of chemotaxis following pre-treatment with IL-4 was mediated by the type I IL-4 receptor because bone marrow-derived eosinophils from γC-deficient mice showed no enhanced migration to eotaxin-1 following IL-4 pre-treatment compared to the wildtype cells. We also show for the first time that mouse eosinophils responded to IL-4 by inducing strong tyrosine phosphorylation of STAT6 and IRS-2. In contrast, IL-13 only induced weak tyrosine phosphorylation of these downstream signaling proteins. ERK and p38 were not phosphorylated in response to IL-4/−13 but there was a slight induction of phospho-AKT^Ser473^ following IL-4 stimulation but less with IL-13.

These findings are important in the context of eosinophilic chemotactic responses in allergic disease. They suggest that eosinophils can become more migratory in the presence of the Th2 cytokine IL-4. This cytokine can also inhibit eosinophil apoptosis [35], stimulate chemokine (MIP-1α and RANTES) synthesis and release of MBP. Therefore, IL-4 priming could augment the infiltration of eosinophils into the lung tissue. Once the eosinophils have migrated into the lung tissue, IL-4 from airway mucosal mast cells and CD4+ T-cells could continue to prime them for migration to the airways. Although the concentrations of cytokines utilized in our study are typical of *in vitro* studies with this cell type, they are above those measured in physiological fluids (BALF, serum) in the setting of allergic inflammation in humans and mice. However, BALF is much diluted upon collection and IL-4 and IL-5 are often unmeasurable in serum, these higher concentrations may be representative of those found at the “allergic synapse” – the close interface between migrating eosinophils and degranulating mast cells, basophils or Th2 cells which produce high levels of the cytokine [36]. IL-4 is believed to be involved in the early phases of the allergic inflammatory response, with IL-13 acting later as an “effector” cytokine. This temporal difference could explain how our data presented here fit with the observation from earlier *in vivo* studies, showing IL-4 secretion into the BALF peaked at 24 hours post-intranasal challenge, followed by an influx of eosinophils into the BALF and lung tissue that peaked at 48 hours [37]. From our experiments comparing the effect of IL-4 and IL-13 on migration and the movement of IL-4-treated WT and γC-deficient eosinophils, we conclude that IL-4, but not IL-13, acting through the type I IL-4 receptor plays a significant role in eosinophil migration. This conclusion is supported by the observation that there was only a minor reduction in eosinophilic inflammation in response to OVA and to IL-4 in the IL-13Rα1-deficient (type II receptor-deficient) mice despite the absence of eosinophilic chemokines [14]. Taken together with our data, these observations point to a direct role for type I IL-4 receptor signaling in eosinophil migration. In light of recent reviews on the subject, our study strengthens the notion of Th2-cytokine cross-talk in both directions between eosinophils and Th2-cells in allergic inflammation [38]–[40].

We observed a difference in the magnitude of tyrosine phosphorylation of STAT6 and IRS-2 following stimulation with IL-4 compared to IL-13. IL-4 elicited robust tyrosine phosphorylation of STAT6 and IRS-2 compared to IL-13 cytokine in these experiments. This difference in intensity of tyrosine phosphorylation is similar to that which we observed in the airway epithelial cell line, A549 [41], in macrophages [12] and other cell types [42], in that IL-4 more potently induced tyrosine phosphorylation of STAT6 (and IRS-2 in macrophages) than IL-13. However, in contrast to macrophages, the difference in STAT6 phosphorylation induced by IL-4 *versus* IL-13 was greater in eosinophils than the difference between IRS-2 tyrosine phosphorylation elicited by these two cytokines. The mouse eosinophils expressed a moderately high amount of IL-13Rα1 on their surface, relative to γC, which can result in IL-13 being a stronger inducer of STAT6 activation compared to IL-4, as was observed in peritoneal *versus* bone marrow-derived macrophages [43]. It is possible that a higher threshold of IL-13Rα1 expression is necessary for the IL-13-induced phospho-STAT6 response to be more robust than the IL-4-induced response, as was observed in the THP-1 cell line [12]. However, even at very high concentrations (100 ng/ml), IL-13 still did not enhance migration to eotaxin-1. Furthermore, in the absence of the type I receptor (γC^−/−^ bmEos), IL-4 pre-treatment did not enhance chemotaxis to eotaxin-1, suggesting that the type II receptor expressed on eosinophils is not able to mediate this response. It is possible that the engagement of type II receptor in eosinophils favors the activation of phosphatases dampening any activating signal. Alternatively, expression of IL-13Rα2, the so-called “decoy” receptor, on mouse eosinophils may be either constitutively high or cytokine-induced to inhibit IL-13 signaling [44]. We did detect expression of mRNA for IL-13Rα2 in mouse eosinophils although it was low relative to expression in the mouse macrophage cell line, RAW 264.7. Cell surface expression of IL-13Rα2 could not be measured due to lack of available anti-mouse IL-13Rα2 antibodies for flow cytometry and PCR detection is a poor predictor of receptor protein expression on the cell surface. The other possibility is that treatment with IL-13 downregulates IL-13Rα1 on mouse eosinophils, as was observed for human eosinophils [23].

Activation of the PI3K pathway, in particular PI3Kγ, is one of the central components of eosinophilic migration in response to chemokines *in vitro* [45] and *in vivo* [46]. IL-4 acting through the IRS-2 adapter protein can lead to activation of p85/p110, and PI3K activity was observed in human eosinophils following stimulation with IL-4 [30]. From the studies of PI3Kγ-deficient macrophages and neutrophils, it is clear that another pathway is contributing to chemotaxis, as there is still some migration of these cells in response to chemokine [47]–[50]. In addition, recent data using siRNA knockdown of PI3Kγ in human eosinophils suggested that PI3Kγ has no role in eotaxin-mediated chemotaxis but it plays a major role in PAF-mediated chemotaxis [51]. In contrast, chemotaxis of mouse eosinophils to eotaxin displayed almost complete dependence upon PI3Kγ [45]. Consistent with this study, we found that wortmannin inhibited the eotaxin-1-induced migration of mouse eosinophils almost completely (data not shown). Thus, the contribution of the different isoforms of PI3K to chemotaxis may depend on the particular chemokine stimulus and appears to differ between human and mouse eosinophils. The activation of different PI3K isoforms might be the point at which the IL-4 and chemokine signaling pathways converge to amplify chemotactic responses of eosinophils *in vivo* during an allergic inflammatory response and we incorporated this hypothesis and our findings into a model (Figure 8). IL-4-induced activation of IRS-2 leads to PI3Kα and Akt activation in FDCP cells, a mouse myeloid progenitor cell line [52]. In this regard, we noted slight induction of serine phosphorylated AKT, a downstream target of p85/110 (Figure 8), following IL-4 stimulation and less AKT serine phosphorylation was observed with IL-13 stimulation. Chemokines such as RANTES, SDF-1 and IL-8 can also activate Akt in a PI3K-dependent manner [53]–[55]. Akt can phosphorylate transcription factors such as NF-κB and can also translocate to the nucleus itself to alter transcription, two mechanisms by which the expression of adhesion molecules may be regulated. IL-4-induced enhancement of eosinophil adherence to endothelial cells and extravasation are well-documented, mainly due to upregulation of endothelial VCAM-1, ICAM-1 and the selectins [56]–[58]. Another point at which the two pathways may converge is at the level of cytoskeletal rearrangement. There is a large literature describing regulation of the Rho GTPase family by PI3K and leucocyte mutants of these family members display deficient migration or alterations in cytoskeletal rearrangement. IL-4 also induces rearrangement of the cytoskeleton in B-cells [59], macrophages [60] and neutrophils [61]. In B-cells, these changes are STAT6-dependent and involve Rac1 and Cdc42 [62], [63]. Another point of convergence of IL-4- and chemokine-activated signaling could be activation of Grb2/Sos/Ras/ERK pathway. IL-4-activated IRS-2 associates with Grb2 [64], [65] and G_i_α-activated Src tyrosine kinases can potentially phosphorylate adaptor proteins such as Shc and Grb2. We however did not observe enhancement of ERK signaling following co-stimulation with IL-4 and eotaxin-1.

In conclusion, we have shown that mouse eosinophils express all components of the IL-4/−13 receptor complexes on their surface, they respond to IL-4 by phosphorylating IRS-2 and STAT6, and their chemotactic responses to eotaxin-1 are enhanced by pre-treatment with IL-4 but not with IL-13. This enhancement of chemotaxis is dependent upon the activation of type I IL-4 receptor complexes. These data suggest that IL-4 in the inflammatory *milieu* would promote increased eosinophilic recruitment into the airways during an allergic immune response. Understanding how cytokine signaling influences chemotactic responses of these cells is important as it might provide a starting point for rational drug design to inhibit inflammatory cell infiltration of the airways in asthma.
